# Engineered bacteria as living therapeutics: Next-generation precision tools for health, industry, environment, and agriculture

**DOI:** 10.3934/microbiol.2025042

**Published:** 2025-12-15

**Authors:** Imen Zalila-Kolsi

**Affiliations:** College of Medical and Health Sciences, Liwa University, Abu Dhabi P.O. Box 41009, United Arab Emirates

**Keywords:** Engineered bacteria, living therapeutics, synthetic biology, CRISPR-Cas systems, microbiome engineering

## Abstract

Synthetic biology has revolutionized precision medicine by enabling the development of engineered bacteria as living therapeutics, dynamic biological systems capable of sensing, responding to, and functioning within complex physiological environments. These microbial platforms offer unprecedented adaptability, allowing for real-time detection of disease signals and targeted therapeutic delivery. This review explores recent innovations in microbial engineering across medical, industrial, environmental, and agricultural domains. Key advances include CRISPR-Cas systems, synthetic gene circuits, and modular plasmid architectures that provide fine-tuned control over microbial behavior and therapeutic output. The integration of computational modeling and machine learning has further accelerated design, optimization, and scalability. Despite these breakthroughs, challenges persist in maintaining genetic stability, ensuring biosafety, and achieving reproducibility in clinical and industrial settings. Ethical and regulatory frameworks are evolving to address dual-use concerns, public perception, and global policy disparities. Looking forward, the convergence of synthetic biology with nanotechnology, materials science, and personalized medicine is paving the way for intelligent, responsive, and sustainable solutions to global health and environmental challenges. Engineered bacteria are poised to become transformative tools not only in disease treatment but also in diagnostics, biomanufacturing, pollution mitigation, and sustainable agriculture.

## 1. Introduction

The combination of synthetic biology and microbiology has initiated a revolutionary phase in therapy, placing modified bacteria as versatile tools for diagnosing, treating, and potentially preventing diseases in the human body. In contrast to conventional drugs, these living therapies can detect environmental signals, react to disease indicators, and perform intricate therapeutic tasks instantaneously [1]–[4].

Recent developments have shown the capability of modified bacteria in different therapeutic scenarios. For example, modified *Escherichia coli* strains have been created to generate anti-inflammatory compounds directly at locations of intestinal inflammation, providing focused therapy for issues such as inflammatory bowel disease [5]. In cancer treatment, non-harmful bacteria have been employed to transport immunostimulatory substances to tumor locations, greatly improving the effectiveness of immunotherapies [6]. These bacteria can migrate to low-oxygen tumor microenvironments and deliver agents that stimulate immune cells, demonstrating their promise as living precision therapies.

The adaptability of engineered bacteria is increasingly evident in the treatment of metabolic disorders. Recent advances have demonstrated that microbes can be genetically modified to convert carbon-rich waste feedstocks into valuable therapeutic proteins, such as insulin analogs and interferon-alpha2a, offering a sustainable and cost-effective approach to drug manufacturing [7]. Complementing these innovations, material science techniques like hydrogel encapsulation have been developed to protect engineered bacteria during transport and extend their functional lifespan within the body. Notably, polyethylene glycol-based hydrogels and hyper-porous encapsulation methods have shown promise in maintaining bacterial viability and enhancing therapeutic efficacy in vivo [8],[9].

Even with these encouraging advancements, the clinical application of engineered bacterial therapies encounters significant obstacles. Guaranteeing biosafety, genetic consistency, and accurate regulation of bacterial function in the intricate human body setting is crucial. Moreover, the regulatory framework for live biotherapeutics is changing, necessitating thorough evaluation of effectiveness, containment measures, and possible off-target impacts [9].

**Figure 1.**
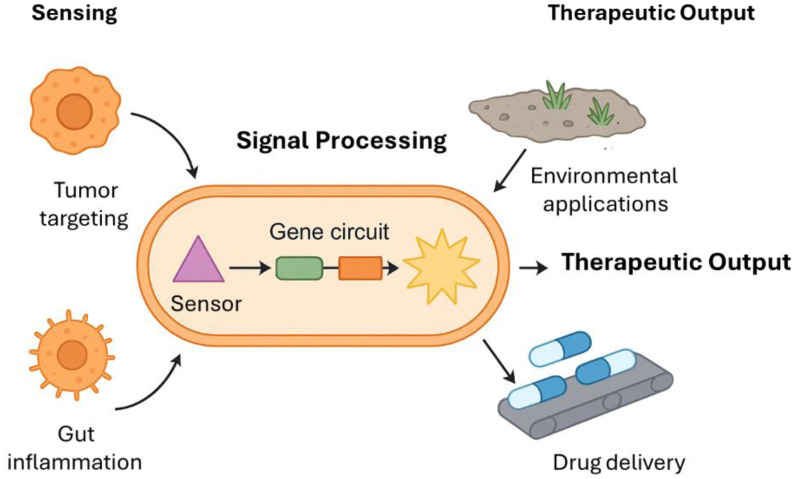
Schematic illustration of engineered bacteria as living therapeutics, showing the flow from sensing disease signals to therapeutic output.

This review offers a thorough summary of the latest developments in modified bacteria as biological treatments. We concentrate on the newest genetic manipulation techniques, transmission approaches, and therapeutic uses and address the current obstacles that need to be addressed to transition these groundbreaking therapies from laboratory to patient care (Figure 1).

## 2. Tools and techniques for engineering bacteria

**Figure 2.**
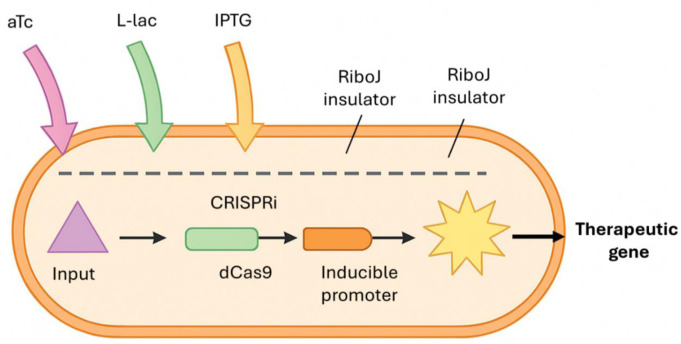
Visual representation of genetic circuit design in engineered bacteria, showing multi-input logic gates, CRISPRi regulation, and inducible promoters.

Advancements in genetic engineering tools have driven the progress of engineered bacteria as living therapies, allowing for accurate and customizable modifications of microbial genomes. The CRISPR-Cas system has evolved into an essential resource for precise gene editing and regulation, enabling scientists to deactivate or stimulate particular genes to adjust bacterial behavior. Prior to the emphasis on CRISPRi, other prevalent CRISPR/Cas methodologies comprise Cas9 for accurate genome modification, Cas3 for extensive DNA deletions, MAD7 as an economical option for commercial uses, and Cascade-based systems for multiplex gene regulation [10]–[12]. For instance, Rong et al. (2024) showcased how CRISPR interference (CRISPRi) can be employed to dynamically regulate metabolic pathways in probiotic strains, improving their therapeutic production while reducing metabolic strain [13]. In addition to this, conventional recombinant DNA technology continues to be essential for creating plasmids and incorporating therapeutic genes. Wu et al. (2024) employed recombinant methods to engineer *E. coli* strains that release anti-inflammatory cytokines in response to gut inflammation signals [14]. Progress in synthetic gene circuits has allowed bacteria to interpret intricate environmental signals and perform logical action: Nguyen et al. (2025) developed a multi-input gene circuit in *Salmonella* that detects tumor-specific microenvironmental markers and activates localized release of immunostimulatory agents, enhancing tumor targeting precision [15] (Figure 2). Additionally, groundbreaking genome editing techniques such as base editing and prime editing, allowing for single-nucleotide changes without inducing double-strand breaks, have been effectively utilized to improve bacterial strain stability and safety [16]. This was demonstrated by Zhang et al. (2024), who made precise modifications to decrease immunogenicity in therapeutic strains [16]. Ultimately, advanced DNA assembly methods like Gibson Assembly and Golden Gate Assembly enable the swift creation of intricate genetic constructs. Taschner et al. (2024) utilized these techniques to construct modular plasmids that encode multi-gene therapeutic payloads effectively, hastening the design-build-test cycle for living therapeutics [17].

## 3. Design principles and computational tools

Recent progress in computational biology has greatly improved the design and enhancement of biological systems. In systems biology, genomic-scale metabolic models (GEMs) are vital instruments for comprehending interactions and metabolic abilities within microbial communities [18]. Hsieh et al. (2024) performed a comparative study of GEMs built with CarveMe, gapseq, and KBase, showing that consensus models combining results from various reconstruction methods provide better functional coverage and fewer dead-end metabolites, thus increasing the predictive capability of community-level metabolic modeling [19]. Simultaneously, machine learning has surfaced as a revolutionary method in strain enhancement. Jin et al. (2025) proposed a framework based on recurrent neural networks for the inverse design of strain fields in hierarchical structures, attaining more than 99% accuracy in forecasting mechanical properties and refining material designs via evolutionary algorithms. Their research emphasizes the scalability and accuracy of machine learning–based design methods for intricate biological systems. Predictive instruments for gene circuit design have advanced, with systems like CLASSIC facilitating high-throughput exploration of genetic design landscapes in mammalian cells, supported by machine learning to anticipate circuit performance [20]. Zhang et al. (2024) advanced the design framework by presenting frequency-controlled gene circuits that can reach new cellular states via dynamic signal modulation. Moreover, Sechkar and Steel (2025) created a model-based strategy to design genetically stable cell groups, utilizing resource-conscious simulations to reduce mutation transmission and improve circuit resilience [21]. These advancements highlight a move toward comprehensive, data-focused approaches that merge mechanistic modeling with machine learning to facilitate accurate and scalable engineering of biological systems.

## 4. Applications for engineered bacteria

### 4.1. Medical applications

In recent times, modified bacteria have achieved significant advancements as drug delivery systems, especially in cancer treatment. For instance, Shen et al. (2024) engineered *Lactobacillus plantarum* to deliver a prodrug that converts into the chemotherapeutic SN-38 specifically within nasopharyngeal cancer tumors. In preclinical models, these bacteria targeted tumors, decreasing tumor growth by approximately 67% and improving drug efficacy by around 54% compared to systemic delivery [22]. A notable study conducted by Yang et al. (2025) developed a nonpathogenic strain of *E. coli* that displays a decoy-resistant interleukin-18 (IL-18) on its surface; the bacteria function as a “tumor GPS”, targeting hypoxic tumor regions and enhancing the activity of CD8⁺ T cells and NK cells, while improving outcomes alongside CAR-NK therapy [23].

Advancements in probiotic engineering for gut health and immune modulation continue. In a research experiment, Tumas et al. (2023) modified *Escherichia coli* Nissle 1917 (EcN) to continuously produce interleukin-2 (IL-2) for the treatment of inflammatory bowel disease following oral delivery; the engineered strain reestablished immune equilibrium and improved disease symptoms in animal models [24]. An additional instance is the research by Woo et al. (2025), who developed inflammation-responsive genetic circuits incorporating a RiboJ insulator (a self-cleaving ribozyme element used to reduce transcriptional interference and improve gene expression consistency) in *E. coli*, allowing for the synthesis of indoleacetic acid solely in inflammatory environments; this reduces off-target impacts and enhances the accuracy of therapeutic effects in the gut [5].

Ultimately, the bacterial detection of pathogens or biomarkers has advanced significantly. A study by Tanniche and Behkam (2023) examined engineered live bacteria employed as tools for disease detection, modifying them to identify biomarkers in situ through genetic logic circuits, providing sensitive and minimally invasive diagnostic options [25]. A recent study by Fdez-Sanromán et al. (2025) created a whole-cell biosensor capable of identifying waterborne pathogens such as *Pseudomonas aeruginosa* and *Burkholderia pseudomallei* by detecting quorum-sensing molecules; once the sensor module is triggered, it produces EGFP in *E. coli*, offering a quick indication of pathogen presence [26].

### 4.2. Industrial and biotechnological applications

Engineered bacteria have greatly advanced industrial biotechnology by producing biofuels, bioplastics, enzymes, and other valuable substances through fermentation or biomanufacturing. Engineered microbes have significantly progressed industrial biotechnology by generating biofuels, bioplastics, enzymes, and various valuable products via fermentation or biomanufacturing. Particularly, bacteria of industrial significance like *Streptomyces* (antibiotics), *Bacillus* (enzymes), and *Corynebacterium* (amino acids) are extensively utilized in large-scale bioprocesses [27]–[29]. Additionally, recent advances highlight the engineering of *Paenibacillus polymyxa* for sustainable applications, ranging from soil health to molecular engineering and medical biotechnology [30]. A significant recent development features *E. coli* engineered to create 1-alkenes (biofuel components) by integrating a chimeric membrane enzyme (UndB combined with catalase) and optimized redox partners; this modified whole-cell biocatalyst attained high specificity and conversion of fatty acids into alkenes with few byproducts, representing progress for sustainable biofuel production [31]. In the realm of bioplastics, Chae et al. (2025) modified *E. coli* to produce polyester amides (PEAs) by utilizing various amino acids and hydroxy acids; they reached production levels of approximately 55 g/L, with polymer characteristics nearing those of traditional plastics, illustrating the practicality and scalability of microbial methods for developing new polymer types [32]. A different study by Riaz et al. (2025) enhanced the production of polyhydroxybutyrate (PHB) in *Bacillus cereus* ARD-03 utilizing agricultural biomass; by process engineering (choice of substrate, pretreatment of biomass, fermentation conditions), they increased the yield and sustainability of bioplastic production from waste feedstocks [33]. In addition to materials, the biomanufacturing of pharmaceuticals and enzymes is experiencing advancements; for instance, systems that integrate microalgae and modified bacteria have demonstrated the ability to generate valuable compounds such as lycopene and reporter proteins, and there is increasing interest in expanding these platforms to include therapeutic proteins [20]. Collectively, these studies demonstrate the increasing capability of engineered bacterial systems to generate industrially significant molecules and materials in a more sustainable manner, although obstacles persist in scaling up, pricing, and maintaining product uniformity.

### 4.3. Environmental applications

Environmental problems such as pollution, increasing CO₂ levels in the atmosphere, and polluted water have emerged as urgent worldwide concerns, with engineered bacteria taking on progressively advanced roles in tackling these issues [34]. A recent study developed living materials from cyanobacteria by incorporating phytochelatin and metallothionein genes into *Synechocystis* sp. PCC 6803; the altered strain exhibited increased resistance to heavy metals (Cd²⁺, Zn²⁺, Cu²⁺) and greater efficiency in removing contaminants from water, illustrating how genetic enhancement can improve both durability and remediation performance [35].

In the field of carbon capture and climate mitigation, scientists have developed “living porous ceramics” by incorporating photosynthetic cyanobacteria and genetically modified *E. coli* into porous ceramics; this composite substance not only absorbs CO₂ from the atmosphere but also detects harmful gases, merging environmental cleanup with biosensing [36].

A different study examined *Gluconobacter oxydans* strains utilized in biomining, highlighting that genes associated with rock weathering and metal extraction additionally aid in improved carbon capture through natural mineral formation; this dual function aids in reducing CO₂ while recovering rare-earth metals [37].

In terms of waste management and water treatment, engineered *Bacillus subtilis* has proven effective in the bioremediation of water contaminated with lead, copper, and cadmium, eliminating notable amounts of these heavy metals under water treatment conditions; this indicates that engineered bacteria are affordable, scalable solutions for purifying industrial effluents [38].

These instances demonstrate how engineered bacteria for environmental use are increasingly multi-functional, incorporating pollutant elimination, climate action, and analytical detection in cohesive solutions.

### 4.4. Agricultural applications

Agriculture is progressively utilizing engineered bacteria to improve plant growth, strengthen disease resistance, and control pests in a more sustainable manner. A notable progress in biofertilizer innovation was achieved with *Geobacter sulfurreducens*, where the removal of the ammonium transporter gene *amtB* resulted in a strain (ΔamtB) exhibiting an unprecedented biological nitrogen fixation rate (~20.6 mg N L⁻¹ day⁻¹), almost twice that of the wild type; this strain successfully enhanced the growth of *Arabidopsis thaliana* in biofertilizer experiments [37]. Likewise, *Rhodopseudomonas palustris* has been modified to produce Fe-only nitrogenase (*anfHDGK*), functioning in the presence of ammonium due to regulation by a constitutively active NifA* regulator. This modified strain enhanced rice growth when applied as a biofertilizer, indicating a potential decrease in reliance on synthetic fertilizers [39].

To broaden the advantages of biofertilizers to non-leguminous plants, research involving *Rhizobium* species revealed that certain isolates enhanced wheat growth as well as nutrient (N and P) absorption across different fertilizer conditions, suggesting synergistic effects of biofertilizer and plant growth promotion [40]. In addition to encouraging growth, engineered microbiomes are utilized to improve disease resistance. Su et al. (2024) increased the expression of a gene in the lignin biosynthesis pathway in rice, changing leaf metabolite production to favor “beneficial” bacteria; this alteration resulted in enhanced resistance to *Xanthomonas oryzae* (bacterial blight) relative to wild-type plants [41].

**Figure 3.**
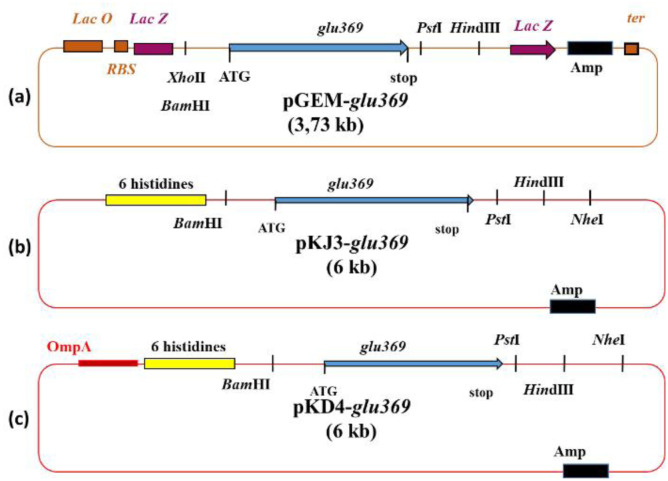
Schematic representation of the recombinant plasmids pGEM-glu369 (a), pKJ3-glu369 (b), and pKD4-glu369 (c) highlighting key genetic elements, including the ampicillin resistance gene (*Amp*), the lacZ gene encoding β-galactosidase, and the OmpA signal sequence [42].

In their 2018 study, Zalila-Kolsi et al. investigated the heterologous expression and periplasmic secretion of an antifungal endo-β-1,3-1,4-glucanase from *Bacillus amyloliquefaciens* BLB369 in *E. coli* Top10. This enzyme plays a crucial role in degrading β-glucan polymers in fungal cell walls, which is vital for combating pathogens like *Fusarium graminearum*, a significant threat to wheat crops [42] (Figure 3).

Ultimately, pest management through bacterial toxins is continually advancing; recent surveys highlight genetically modified entomopathogenic bacteria (GM-EPBs) like *Bacillus*, *Pseudomonas*, *Serratia*, *Photorhabdus*, and *Xenorhabdus*, designed for improved toxin yield and increased field resilience [43].

Beyond these examples, other species like *Bacillus subtilis* 7611 and *Klebsiella pneumoniae* DSM 30104 have been thoroughly studied for use in agriculture. Modified *B. subtilis* 7611 strains featuring improved regulatory pathways and substituted promoters can generate excess lipopeptides such as surfactin and fengycin, boosting biocontrol effectiveness and promoting plant development [44]. In a similar manner, *K. pneumoniae* DSM 30104 has been engineered to enhance nitrogen fixation effectiveness through the optimization of *nif* gene expression and regulatory pathways, thereby aiding soil fertility and decreasing reliance on synthetic fertilizers. Importantly, DSM 30104 is a nonpathogenic strain used under controlled conditions, and genetic attenuation combined with containment strategies ensures its safe application in agricultural environments [45]. These enhancements highlight the growing significance of engineered bacteria in sustainable farming, extending past conventional biofertilizers to encompass strains tailored for improved biocontrol, nutrient uptake, and stress tolerance.

**Table 1.** Recent applications of engineered bacterial strains across therapeutic, environmental, industrial, and agricultural domains, highlighting their targets and mechanisms of action.

Application	Engineered bacteria	Target	Mechanism	Ref
Therapeutic	*E. coli* Nissle 1917	Inflammatory bowel disease (IBD)	IL-2 production: Engineered *E. coli* Nissle 1917 secretes interleukin-2 to modulate immune response and reduce inflammation in IBD.	[24]
	*Lactobacillus plantarum* WCFS1	Nasopharyngeal cancer	Prodrug activation (SN-38): *L. plantarum* converts inactive SN-38 prodrug into its active form at tumor sites, enhancing localized cancer therapy.	[22]
	*E. coli* K-12 DH5α	Hypoxic tumors	IL-18 surface display: *E. coli* displays IL-18 on its surface to stimulate immune cells and target hypoxic tumor microenvironments.	[23]
Environmental	*Synechocystis* sp. PCC 6803	Heavy metals (Cd²⁺, Zn²⁺, Cu²⁺)	Phytochelatin and metallothionein expression: Synechocystis sp. PCC 6803 expresses metal-binding peptides to sequester and detoxify heavy metals.	[35]
	*Bacillus subtilis* 7611	Lead, copper, cadmium	Bioremediation: *Bacillus subtilis* metabolizes and immobilizes heavy metals like lead and copper, aiding in environmental cleanup.	[38]
Industrial	*E. coli* BL21(DE3)	Biofuel production	1-alkene synthesis via UndB enzyme: *E. coli* is engineered to express UndB enzyme for converting fatty acids into industrially useful alkenes.	[31]
	*E. coli* XL1-Blue	Bioplastics	PEA production from amino/hydroxy acids: *E. coli* synthesizes biodegradable polyesters using amino and hydroxy acid precursors.	[32]
Agricultural	*Geobacter sulfurreducens* PCA	Nitrogen fixation	ΔamtB strain with enhanced fixation rate: Geobacter sulfurreducens with amtB deletion shows improved nitrogen fixation for soil enrichment.	[37]
	*Rhizobium* sp.	Wheat growth	Nutrient absorption enhancement: *Rhizobium* sp. enhances nutrient uptake in wheat roots, promoting growth and yield.	[40]
	*E. coli* Top 10	Antifungal activity	β-glucanase secretion against *Fusarium*: *E. coli* secretes β-glucanase enzyme to degrade fungal cell walls and protect crops from *Fusarium* infection.	[42]
	*Bacillus subtilis 7611*	Biocontrol and plant growth promotion	Engineered *Bacillus subtilis* strain with optimized regulatory pathways and promoter replacements to overexpress surfactin and fengycin operons, enhancing pathogen suppression and promoting plant growth.	[44]
	*Klebsiella pneumoniae DSM 30104*	Nitrogen fixation and soil fertility improvement	Enhanced nitrogen fixation through optimization of *nif* gene expression and regulatory pathways to improve ammonium assimilation and crop growth.	[45]

## 5. Ethical, safety, and regulatory considerations

As synthetic biology approaches practical application, clear ethical, safety, and regulatory frameworks are essential to support responsible innovation. Containment strategies such as kill-switches (genetic circuits designed to trigger cell death under specific conditions) and auxotrophy (a condition where an organism cannot synthesize an essential compound and must obtain it externally) are being refined to prevent the unintended release of genetically modified organisms (GMOs) into the environment. Rottinghaus et al. (2022) demonstrated the rational design of evolutionarily stable microbial kill switches using toxin-antitoxin systems and CRISPR-based constructs, showing long-term functionality over 140 generations in *E. coli* [46]. These considerations are essential as engineered bacteria transition from laboratory research to clinical and environmental deployment.

Public perception and regulatory approaches to GMOs vary significantly across regions and remain subjects of ongoing debate. In 2025, over 200 European organizations, including NGOs, agricultural unions, and organic farming advocates, issued a joint statement opposing the European Commission's proposal to ease regulations on new GMOs. Their concerns included biopiracy, legal uncertainty for breeders, and reduced seed diversity, reflecting broader societal anxieties about transparency, health risks, and corporate influence. While this statement is not yet covered in peer-reviewed literature, Peri et al. (2025) analyzed stakeholder responses to EU consultations on New Genomic Techniques (NGTs), revealing tensions between sustainability goals and regulatory caution, and emphasizing the importance of transparency and risk assessment in shaping public trust [47].

Beyond Europe, various regions have established guidelines to direct synthetic biology and live biotherapeutics. In the United States, the Food and Drug Administration (FDA) offers recommendations for early-phase clinical trials of live biotherapeutic products, emphasizing strain characterization and production regulations [48]. Likewise, the National Institutes of Health (NIH) regulates studies involving recombinant or synthetic nucleic acids through its biosafety regulations [49]. In Asia, Japan oversees genetically modified organisms through the Cartagena Act, mandating containment protocols for both industrial and clinical applications [50], while China has implemented measures like Shenzhen's synthetic biology regulations to foster innovation alongside biosecurity standards [51]. These efforts illustrate a worldwide movement to align safety and innovation in synthetic biology.

Concerns about dual-use and biosecurity are being increasingly managed through strong legal frameworks. Regulation (EU) 2021/821 governs the export, brokering, and transfer of dual-use goods, including biotechnologies such as CRISPR and tools for pathogen research. Johnson et al. (2025) reviewed the evolving regulatory landscape for synthetic biology, emphasizing the importance of frameworks that balance innovation with security. Their analysis highlights key features such as catch-all clauses designed to prevent misuse, human rights considerations embedded in oversight mechanisms, and the implementation of internal compliance programs. These regulatory tools aim to ensure that synthetic biology technologies are developed and deployed responsibly, fostering scientific progress while mitigating potential risks [52].

## 6. Challenges and limitations

Despite the significant advancements in synthetic biology, various ongoing challenges remain that hinder its wider implementation. A major constraint is the stability of engineered traits, which are frequently compromised by spontaneous mutations during cellular growth and division [53].

Recent modeling frameworks have begun to address this issue by linking DNA design to mutation dynamics, allowing forecasts of how synthetic constructs evolve over time in expanding populations. These models incorporate host-sensitive parameters and mutation heterogeneity, offering insights into enhancing genetic longevity and protein production. Ingram et al. (2023) developed a mutation-aware, host-aware framework that connects synthetic DNA design to mutation spread, enabling predictions of protein yield and genetic shelf life in engineered populations. Nonetheless, evolutionary pressures often lead to the loss of synthetic functions, especially in large-scale bioreactors [53].

A significant issue is horizontal gene transfer (HGT), which presents biosafety risks by enabling the unintended dissemination of transgenic DNA across species boundaries. HGT mechanisms, such as transformation, conjugation, and transduction, can facilitate the spread of antibiotic resistance genes and pathogenic traits. Alamnie et al. (2020) emphasized that recombinant DNA from GMOs may be transferred to environmental microbes, potentially contributing to the spread of resistance and virulence factors, and called for stricter biosafety regulations [54].

Moreover, scale-up and reproducibility continue to pose significant challenges. While synthetic biology has demonstrated success in controlled laboratory settings, translating these results to industrial scales often results in reduced yield, inconsistent performance, and increased costs. Furthermore, factors like genetic tractability, morphological instability, and other strain-specific constraints greatly affect scalability and reproducibility. These elements frequently complicate the design-build-test process and necessitate customized approaches to guarantee reliability in industrial and clinical settings [29]. Factors such as environmental sensitivity, biological variability, and infrastructure limitations contribute to these issues. Lux et al. (2023) argued that reproducibility remains a central obstacle in synthetic biology and that systemic changes in education, infrastructure, and incentives are essential to ensure a reliable transition from lab to market [55].

## 7. Future perspectives and emerging trends

Synthetic biology is entering a groundbreaking phase, with new applications that are set to transform medicine, materials science, and microbial ecology. A highly promising avenue is the creation of living therapeutics and “intelligent” bacteria, which are designed to independently detect disease-specific signals and react with precise therapeutic measures [43]–[45]. These smart microbes incorporate biosensing components, signal transduction pathways, and output systems to identify and address issues like cancer, metabolic diseases, and infections directly, frequently alongside nanomaterials and artificial intelligence [56]. Alongside this, the design of microbial consortia and synthetic communities provides a scalable approach to overcoming the constraints of monoculture systems. Consortia facilitate labor division, increase circuit stability, and enhance biosynthetic efficiency by spreading metabolic and regulatory roles among various strains. Techniques like quorum sensing, spatial separation, and ecological interaction modeling are employed to sustain functional equilibrium and avert population decline [57].

The combination of synthetic biology with nanotechnology and materials science represents a new frontier. For example, Kang et al. (2025) in *Cell Biomaterials* demonstrated how heterojunction-functionalized microbial cells can overcome immune suppression and enable catalytic and immunotherapeutic functions [41]. Similarly, Cai et al. (2025) in *Advanced Materials* introduced SynBioNanoDesign platforms that integrate engineered microorganisms with smart nanomaterials, enabling responsive drug delivery and biosensing functionalities [58]. In addition, Nguyen et al. (2023) in *Nature Communications* reported breakthroughs in multi-input genetic circuits and tumor-targeting strategies [59]. These systems reinvent biological elements as programmable substances, allowing for the development of nanomaterials with customized shapes and functions for precise drug delivery, biosensing, and regenerative medicine [58].

Ultimately, microbiome engineering for personalized medicine is gaining traction, utilizing synthetic biology tools such as CRISPR and metabolic pathway design to customize microbial communities based on individual health profiles. Designed probiotics and biosensors are being created to assess and adjust gut health, providing innovative solutions for issues associated with microbiota imbalances and leading to targeted treatments [60]. Collectively, these trends indicate a transformative change toward intelligent, adaptive, and environmentally integrated approaches in synthetic biology.

## 8. Conclusions

Synthetic biology has swiftly progressed into a multifaceted domain that combines computational modeling, machine learning, and systems biology to create and enhance biological systems with unmatched accuracy. Significant progress encompasses the creation of genome-scale metabolic models and tools for predictive gene circuit design, facilitating strategic strain engineering and dynamic regulation of cellular behavior. Reinforcement learning and generative models in machine learning are currently utilized to address the challenges of conventional design-build-test cycles, while strategies like kill-switches and auxotrophy enhance biosafety and adherence to regulations. The sector is also tackling vital issues like trait consistency, horizontal gene transfer, and reproducibility, particularly in large-scale industrial applications. In the future, synthetic biology is set to transform medicine and biotechnology via living therapeutics, engineered microbial communities, and personalized treatments based on the microbiome. The combination of nanotechnology and materials science will enhance its application in intelligent biomaterials and adaptive systems. Future studies will probably concentrate on improving the evolutionary resilience of designed organisms, establishing AI-powered biofoundries, and forming environmentally conscious synthetic communities. As the discipline develops, ethical governance, public involvement, and cross-disciplinary collaboration will be crucial to guarantee responsible innovation and societal advantage.

## Use of AI tools declaration

The authors declare they have not used Artificial Intelligence (AI) tools in the creation of this article.
